# An initial audit – a step in the journey to improve admission medication reconciliation compliance and accuracy for admitted psychiatric patients

**DOI:** 10.1192/j.eurpsy.2026.11711

**Published:** 2026-07-31

**Authors:** A. Raji, J. Crowdis, G. Asurappulige, S. Singh, A. Sharma, J. Ali, Z. Shullaih, I. Hussein, M. Hussain, S. Singh, T. Diepriye, M. A. Mahmoud, F. Rahman, W. DeCoste, B. Foley

**Affiliations:** 1 Psychiatry, Dalhousie University, Halifax; 2 Psychiatry; 3 Pharmacy, Cape Breton Regional Hospital, Sydney; 4 Psychiatry, St. Martha’s Regional Hospital; 5 Quality Improvement & Patient Safety, Nova Scotia Health -Eastern Zone, Antigonish, Canada

## Abstract

**Introduction:**

Medication reconciliation (MedRec) is recognized as an important safety initiative by the World Health Organization (Accreditation Canada, ROP 2020 handbook). An initial audit at a Canadian Regional Hospital acute psychiatric unit revealed that 21/22 (95%) of inpatients had a documented admission Best Possible Medication History (BPMH). 15/21 (71%) contained at least one discrepancy, the most common type being omissions (10/17 total discrepancies, 59%). Documentation of ≥2 MedRec information sources was 4.8% (1/21). Stuijt et al. reported that 36–95% of admissions across six hospitals in the Netherlands had at least one MedRec discrepancy.

**Objectives:**

To improve the completeness, quality, and accuracy of admission MedRecs communicated to care providers for admitted psychiatric patients.

**Methods:**

A quality improvement (QI) project was initiated with an initial audit, of all 22 patients on admission in a regional hospital acute psychiatric unit on a randomly selected day in June 2025. Data sources included admission MedRec forms and medical records. Primary process measures were proportion of patients with a documented MedRec; proportion of admission MedRec with documented ≥2 information sources (including patient/family/caregiver interviews), and the types and frequency of MedRec discrepancies. Prior to late September 2025, BPMHs were typically documented at the hospital using forms that did not include pre-populated provincial Drug Information System (DIS) medication lists. In late September 2025, a DIS MedRec form with a pre-populated DIS pre-admission prescribed medication list was implemented. Clinical team members received education on its use prior to the intervention’s implementation. Pre- and post-intervention data (June – August 2025 vs. November 2025 – January 2026) will be collected, and analyzed using run charts to identify trends or shifts in documentation rates, the frequency of documentation of at least two information sources, and discrepancy frequency.

**Results:**

Initial audit findings were shared with clinicians to highlight gaps in MedRec practices. Pre- and post-intervention data collection and analyses are ongoing to assess expected outcomes – increase in documentation rates, higher use of multiple information sources, and a reduction in MedRec discrepancies.

**Conclusions:**

This audit represents an important first step towards improving MedRec accuracy and quality for psychiatric admissions at the hospital. Results will inform whether additional interventions or process refinements are necessary to achieve and sustain MedRec best practices at the hospital.

**Disclosure of Interest:**

None Declared

